# Improving Ventilator Care Quality Through a Structured Ventilator Pathway: A Retrospective Pre-Post Quality Improvement Study

**DOI:** 10.7759/cureus.108484

**Published:** 2026-05-08

**Authors:** Darshana Rathod, Kankana Samanta, Piyali Chaudhari, Gauri Pathare, Shreyans Rai, Mayur Patel

**Affiliations:** 1 Department of Critical Care Medicine, Sir H. N. Reliance Foundation Hospital and Research Centre, Mumbai, IND; 2 Sir H. N. Medical Research Society, Sir H. N. Reliance Foundation Hospital and Research Centre, Mumbai, IND; 3 Department of Academics and Research, Sir H. N. Reliance Foundation Hospital and Research Centre, Mumbai, IND

**Keywords:** intensive care unit, mechanical ventilation, prevention bundles, quality of care, ventilator-associated events

## Abstract

Introduction

Introduction

Mechanical ventilation (MV) is a lifesaving intervention for critically ill patients; however, it is associated with increased risk of developing mechanical, pulmonary, and sepsis-related complications, as well as high mortality. The ventilator-associated pneumonia (VAP) bundle, though widely used, is limited by its subjectivity and poor sensitivity and specificity. The National Healthcare Safety Network introduced ventilator-associated events (VAE) surveillance in 2013, subsequently modified by the Centers for Disease Control and Prevention (CDC), with an updated VAE classification: ventilator-associated condition (VAC), infection-related ventilator-associated complication (IVAC), and possible ventilator-associated pneumonia (PVAP). This study aimed to assess VAE incidence at a tertiary care center and evaluate the impact of implementing a ventilator pathway (an educational and interventional initiative) on patient outcomes and VAE rates.

Methods

This retrospective study included patients receiving MV for >48 hours. A pre-implementation survey, conducted among intensive care unit (ICU) staff (n=140) regarding components of ventilator care, along with an audit of VAP bundle awareness/compliance, identified deficiencies in ventilator care practices, lack of monitoring, and gaps in knowledge. In response, a ventilator pathway incorporating evidence-based care components was developed, followed by staff training and implementation. VAE was identified using CDC-defined criteria.

Results

The study population was divided into two groups: pre-implementation (n=137) and post-implementation (n=431). Baseline characteristics and disease severity were comparable between the groups. Hospital length of stay decreased from 19 to 16 days (p=0.01), with no significant differences in ICU length of stay or duration of MV. Survival was higher in the post-implementation group (63.1% (272/431)) compared to that in the pre-implementation group (56.2% (77/137)), although the difference was not statistically significant. VAE incidence decreased significantly from 28.46% (39/137) to 3.71% (16/431) (p<0.001), corresponding to an 87% relative risk reduction. Similar significant decreases were observed in the incidences of VAC (15/137 (10.94%) vs. 11/431 (2.55%); p<0.001), IVAC (7/137 (5.11%) vs. 6/431 (1.39%); p<0.05), and PVAP post-implementation of the pathway (10/137 (7.29%) vs. 1/431 (0.23%); p<0.001). Total infection rate decreased from 12.40% (17/137) to 1.62% (7/431) (p<0.001) after pathway implementation.

Conclusion

Implementation of a structured ventilator pathway, combined with targeted education and adherence monitoring, was associated with reduced VAE incidence and infection rates and improved patient outcomes. These findings indicate improved infection control and care standardization after pathway implementation. These improvements highlight the effectiveness of clinical pathways as sustainable quality improvement tools.

## Introduction

Ventilator-associated pneumonia (VAP) is one of the most severe and frequent nosocomial infections in the intensive care unit (ICU). Its development is directly associated with prolonged mechanical ventilation (MV), extended ICU stay, and higher mortality. According to the U.S. Centers for Disease Control and Prevention (CDC), ventilator-associated respiratory infections (VARI) are the most frequent complications of MV. Therefore, accurate identification and management of VAP are critical in improving outcomes for mechanically ventilated patients [1,2].

Traditionally, VAP diagnosis relied on clinical signs (fever, worsening oxygenation, changes in secretions), chest radiography, and microbiological data. However, these diagnostic criteria are often subjective, variable, and lack consistency. In response to these limitations, the CDC introduced the ventilator-associated event (VAE) surveillance framework in 2013, which uses a broader, more objective, and standardized algorithm to capture a wider range of complications associated with MV, including both infectious and non-infectious events. VAP has been identified as VAE by the CDC since 2013, emphasizing a shift from subjective diagnosis toward standardized event detection [3].

The CDC classifies VAEs into three tiers: ventilator-associated condition (VAC), infection-related ventilator-associated complication (IVAC), and possible ventilator-associated pneumonia (PVAP) [3,4]. Within the CDC surveillance framework, VAC is defined as a sustained worsening of oxygenation following a baseline period of stability or post-MV improvement. VAC captures both infectious and non-infectious causes of respiratory decline such as atelectasis, pulmonary edema, and acute respiratory distress syndrome (ARDS). IVAC is identified as VAC accompanied by signs of infection (abnormal temperature or white blood cell count) and ≥4 days of new antimicrobial agent administration; this requirement increases the specificity and clinical credibility of the definition [5]. PVAP is diagnosed when IVAC criteria are met in addition to microbiological evidence, such as purulent respiratory secretions, positive cultures, or, less commonly, histopathological confirmation, pleural fluid culture, or specific viral or Legionella testing [6].

VAE-related infections are associated with antibiotic resistance, prolonged hospitalization, increased healthcare costs, and higher mortality and morbidity. Thus, their prevention is crucial in mitigating adverse patient outcomes and lowering the overall burden on healthcare systems. VAP is commonly caused by a range of hospital-acquired pathogens, and understanding the microbiological profile of VAP is important when considering prevention strategies as well as the limitations of infection-focused surveillance. Common VAP pathogens, particularly in patients requiring prolonged MV, include *Pseudomonas aeruginosa*, *Escherichia coli*, *Klebsiella pneumoniae*, Gram-negative *Acinetobacter* species, and Gram-positive *Staphylococcus aureus*. Although VAP diagnostic criteria may vary across institutions, they typically include leucocytosis, fever, progressive infiltration on chest X-ray, positive respiratory secretion cultures, and decreased gas exchange [7]. However, diagnosing lung infections in mechanically ventilated patients remains challenging, despite advances in surveillance definitions and frameworks [8]. Therefore, identifying effective, reproducible strategies to prevent both VAP and broader VAEs has become a major research focus in recent years [9]. Implementation of VAP care bundles has demonstrated effectiveness in reducing VAP incidence, particularly when paired with specific training programs to improve staff compliance [10]. However, focusing exclusively on VAP may underestimate the total burden of ventilator-related complications, and factors influencing the risk of VAEs should guide the implementation of ventilator care bundles [11]. 

Although VAEs have been linked to specific patient populations, such as trauma cohorts [12], their independent association with ICU outcomes, including mortality, remains uncertain [13,14]. Nevertheless, VAE episodes have been associated with longer ICU stay and prolonged duration of MV [13]. Prior to the current CDC surveillance paradigm, VAP-attributable mortality in trauma patients was considered negligible, despite VAP being the most common nosocomial infection in this group and being associated with extended ventilation, systemic infectious complications, higher costs, and lower discharge rates [15,16]. Given evolving definitions, limitations of traditional VAP surveillance, and the recognized impact of VAEs on patient outcomes, there is a growing need for standardized, evidence-based approaches to ventilator care that emphasize prevention, early detection, and consistent practice across ICU teams. Clinical pathways represent one such approach, offering structured guidance, education, and monitoring to improve adherence to best practices and reduce preventable complications.

This study aimed to evaluate VAE incidence in the ICU of a tertiary healthcare center and assess the impact of implementing a structured ventilator pathway on infection rates, mortality, and patient outcomes. The primary objective was to evaluate the change in VAE incidence following pathway implementation. Secondary objectives included assessment of hospital length of stay, ICU length of stay, duration of mechanical ventilation, survival, infection rates, compliance with ventilator care practices, and changes in the distribution of VAC, IVAC, and PVAP.

## Materials and methods

Study design

A retrospective cohort study was conducted at Sir H. N. Reliance Foundation Hospital and Research Centre (2020-2023) to evaluate VAE incidences before and after the implementation of a structured ventilator pathway in the ICU. The pathway was implemented in January 2022. The project included a pre-intervention survey assessing knowledge gaps among ICU clinicians, followed by the development and implementation of a ventilator pathway and subsequent monitoring of compliance and outcomes.

The study adhered to the CDC guidelines for the electronic identification of VAEs and was conducted in accordance with the Declaration of Helsinki. It was approved by the Institutional Ethics Committee (HNH/IEC/2023/OCS/CCM/119), and the requirement for informed consent was waived owing to its retrospective observational design.

Study participants and analytical framework

Adult (≥18 years) medical and surgical patients admitted to the ICU who required invasive MV for ≥48 consecutive hours were included, in accordance with CDC VAE surveillance criteria [17]. Patients undergoing tracheostomy and those requiring reintubation during the ICU stay were included, provided they met the duration criterion. Patients intubated and receiving mechanical ventilation prior to transfer from other healthcare facilities were excluded.

The study followed a PECO (Population, Exposure, Comparator, Outcome) framework. The population comprised adult ICU patients receiving MV. The exposure was invasive MV for ≥48 hours. The comparator was the pre- versus post-implementation period of the ventilator pathway. The primary outcome was VAE incidence (VAC, IVAC, PVAP). Secondary outcomes included evaluation of associations between patient demographics, comorbidities, and VAE development.

Study variables

Demographic and clinical variables included age, sex, comorbidities, admission diagnosis, ICU and hospital length of stay (LOS), duration of MV, and disease severity as assessed by the Acute Physiology and Chronic Health Evaluation II (APACHE II) score. The APACHE II score was calculated using 12 physiological variables recorded within the first 24 hours of ICU admission, along with age and chronic health status; higher APACHE II scores indicate greater severity of illness and are associated with increased risk of mortality [18].

Ventilator pathway development and implementation

A pre-implementation survey of ICU physicians and nurses identified knowledge and compliance gaps in key components of the ventilator care bundle, including delirium assessment, spontaneous breathing trial (SBT), spontaneous awakening trial (SAT), and Richmond Agitation Sedation Scale (RASS). The survey included structured questions assessing both awareness and adherence to these components. The survey findings informed the development of a structured educational and interventional program aimed at reducing VAE incidence.

Educational Component

A structured bedside training program was implemented to enhance compliance with ventilator care bundle components. The respiratory therapist in charge conducted daily bedside educational sessions for nurses and physicians. Compliance was tracked using an outcome tool and scorecard. Nursing staff performed daily delirium (Confusion Assessment Method for the ICU (CAM-ICU) score) [19] and sedation (RAAS) [20] assessments using a system-based software. Eligible patients underwent daily SBT and SAT using a standardized weaning checklist. Non-compliance prompted immediate bedside reinforcement and, when required, additional focused educational sessions. A key challenge encountered during implementation was the frequent staff rotation, necessitating repeated training to ensure consistency in knowledge and practice across the ICU team.

VAE Pathway

**Figure 1:**
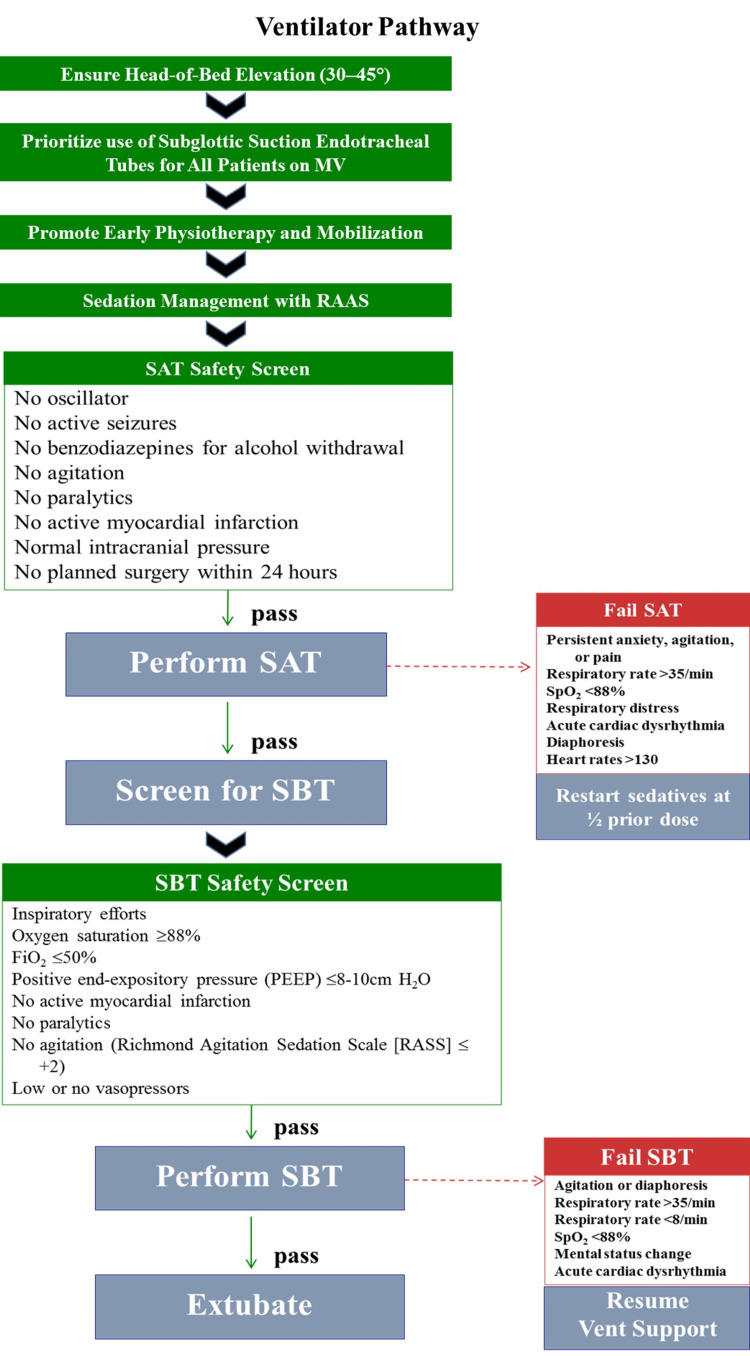
Ventilator pathway This figure illustrates the structured ventilator care pathway, integrating evidence-based practices with defined safety screening and failure pathways. Image credits: Created by the authors using Microsoft PowerPoint (Microsoft Corporation, Redmond, WA, US), based on standard ventilator care bundle practices and AHRQ-recommended SAT-SBT protocols. ETT, endotracheal tube; FiO₂, fraction of inspired oxygen; HOB, head-of-bed; MV, mechanical ventilation; PEEP, positive end-expiratory pressure; RASS, Richmond Agitation Sedation Scale; SAT, spontaneous awakening trial; SBT, spontaneous breathing trial; AHRQ: Agency for Healthcare Research and Quality

A standardized ventilator care pathway (Figure 1) was designed, based on the Agency for Healthcare Research and Quality (AHRQ) guidelines [21], and implemented in the ICU. This pathway served as the central framework for improving compliance and reducing VAE rates. The pathway incorporated the following evidence-based components: (1) head-of-bed elevation between 30° and 45° to reduce aspiration risk; (2) preferential use of endotracheal tubes (ETTs) with subglottic suction ports for patients expected to require >72 hours of MV, as continuous secretion drainage is associated with reduced early-onset VAP; (3) early physiotherapy and mobilization to reduce deconditioning and improve weaning success; (4) light sedation (titrated using RASS) with daily sedation interruptions (“sedation vacations”) to assess neurological function (consciousness level) and weaning readiness; (5) standardized SAT safety screening, followed by SBT; (6) standardized SBT protocol to evaluate extubation readiness; and (7) daily delirium assessments (CAM-ICU tool) for early detection and management, with long-term goals of implementing a comprehensive ICU delirium prevention bundle.

Daily Compliance Monitoring

Compliance with all components of the ventilator pathway was assessed daily by respiratory therapists during ICU rounds for all mechanically ventilated patients. The monitored parameters included hospital and ICU admission date, intubation date and location, diagnosis, presence of subglottic suction, head-of-bed elevation (30-45°), RASS, daily mobilization score, physiotherapy sessions, occurrence of mobility events, CAM-ICU score, and performance of SAT and SBT (including sedation cessation). Compliance was quantified as the proportion of eligible ventilator pathway components completed per patient per day and tracked using a standardized scorecard.

Data collection

Data were extracted from the electronic medical records (EMRs) of the hospital. Adherence to the ventilator pathway was monitored using a digital ventilator outcome tool, and VAE incidence was calculated using the CDC National Healthcare Safety Network (NHSN) surveillance definitions and the CDC VAE calculator [22]. Trends in VAE rates and SAT/SBT performance were analyzed over time to evaluate the impact of the intervention.

Statistical analysis

Normality was assessed using the Shapiro-Wilk test. Continuous variables are presented as mean ± standard deviation (SD) or median with interquartile range (IQR), as appropriate, and compared using the Mann-Whitney U test. Categorical variables, presented as counts and percentages, were compared using the chi-square (𝜒2) test or Fisher's exact test (when >20% of cells had expected frequencies <5). A two-tailed p<0.05 was considered statistically significant. Analyses were performed using Stata version 17 (StataCorp LLC, College Station, TX, USA). Outcomes were analyzed at the patient level rather than per ventilator-days, consistent with the study objectives.

## Results

Patient demographics and comorbidities

A total of 568 patients admitted to the ICU between 2020 and 2023 were included in the study. The study population was divided into two groups based on implementation of the ventilator pathway: pre-implementation (2020-2021, n=137) and post-implementation (2022-2023, n=431). The study cohort had a mean age of 60±15.52 years, with 177 (31.16%) females and 391 (68.84%) males. Approximately 60% of admissions were surgical, with no significant difference in admission type between the two groups.

**Figure 2:**
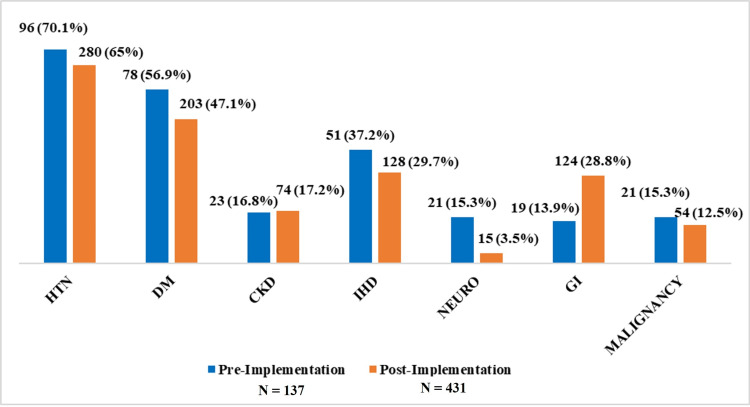
Distribution of baseline comorbidities in pre- and post-implementation cohorts The distribution of baseline comorbidities in pre- (N=137) and post-implementation (N=431) groups are presented as numbers (percentages). No significant differences were observed between the groups. CKD, chronic kidney disease; DM, diabetes mellitus; GI, gastrointestinal; IHD, ischemic heart disease; NEURO, neurological compilations

Figure 2 shows the distribution of comorbidities in the two groups. The most common comorbidities were hypertension and diabetes mellitus in both groups. Other comorbidities included ischemic heart disease, gastrointestinal disorders, malignancy, and chronic kidney disease. There was no significant association between comorbidities and study groups.

Baseline severity and hospitalization outcomes

The median APACHE II scores were comparable between the two groups, with no statistically significant difference. The hospital LOS decreased significantly after implementation of the ventilator pathway (median 19 days vs 16 days, p=0.01). However, the ICU LOS and duration of MV did not significantly differ between the pre-implementation and post-implementation groups (Table 1).

**Table 1:** Severity scores and hospitalization duration outcomes pre- and post-implementation of ventilator pathway Data are presented as median with interquartile range (IQR). P-values were calculated using the Mann-Whitney U test. APACHE II: Acute Physiology and Chronic Health Evaluation II; LOS, length of stay; MV, mechanical ventilation

Covariates	Pre-implementation ( N=137)	Post-implementation ( N=431)	p-value	U Statistic
	Median (IQR)	Median (IQR)		
APACHE II Score	18 (11-23)	18 (13-24)	0.48	28354
Hospital LOS (days)	19 (11-29)	16 (9.5-24)	0.01	25400
ICU LOS (days)	13 (7-23.3)	12 (8-19)	0.14	26847
Duration of MV (days)	8 (4-14)	7 (5-11)	0.42	28169

Patient outcomes and VAE incidences

Survival increased from 56.2% pre-implementation to 63.1% post-implementation of the ventilator pathway; however, the difference was not statistically significant. VAE incidence decreased significantly from 28.46% to 3.71% (p<0.001), corresponding to a relative risk of 0.13 (95% CI: 0.08-0.22), representing an 87% relative risk reduction and an absolute risk reduction of 24.75%; this suggests a strong association with pathway implementation. Significant reductions in VAC (p<0.001), IVAC (p=0.019), and PVAP (p<0.001) incidences were observed following pathway implementation, along with a significant decline in overall infection rates (12.40% vs 1.62%, p<0.001) (Table 2). Across the cohort, the most common VAE etiologies were septic shock (7.92%), sepsis (4.92%), ARDS (3.16%), pneumonia (2.99%), lung collapse (2.28%), hypoxia (0.35%), pneumothorax (0.17%), and alveolar hemorrhage (0.17%).

**Table 2:** VAE and infection incidences pre- and post-implementation of ventilator pathway Values are presented as frequency (%). Comparisons between pre-implementation and post-implementation groups were performed using the chi-square test or Fisher’s exact test, as appropriate; Fisher’s exact test was applied for IVAC and PVAP due to low expected cell counts. p<0.05 was considered statistically significant. VAE, ventilator-associated event; VAC, ventilator-associated condition; IVAC, infection-related ventilator-associated complication; PVAP, possible ventilator-associated pneumonia

Event	Pre-implementation (n=137)	Post-implementation (n=431)	p-value	χ² value
	Frequency (%)	Frequency (%)		
VAE	39 (28.46)	16 (3.71)	<0.001	70.04
VAC	15 (10.94)	11 (2.55)	<0.001	14.91
IVAC	7 (5.11)	6 (1.39)	0.019	-
PVAP	10 (7.29)	1 (0.23)	<0.001	-
Total infection	17 (12.40)	7 (1.62)	<0.001	27.27

Pre-implementation assessment of ventilator care practices

**Figure 3:**
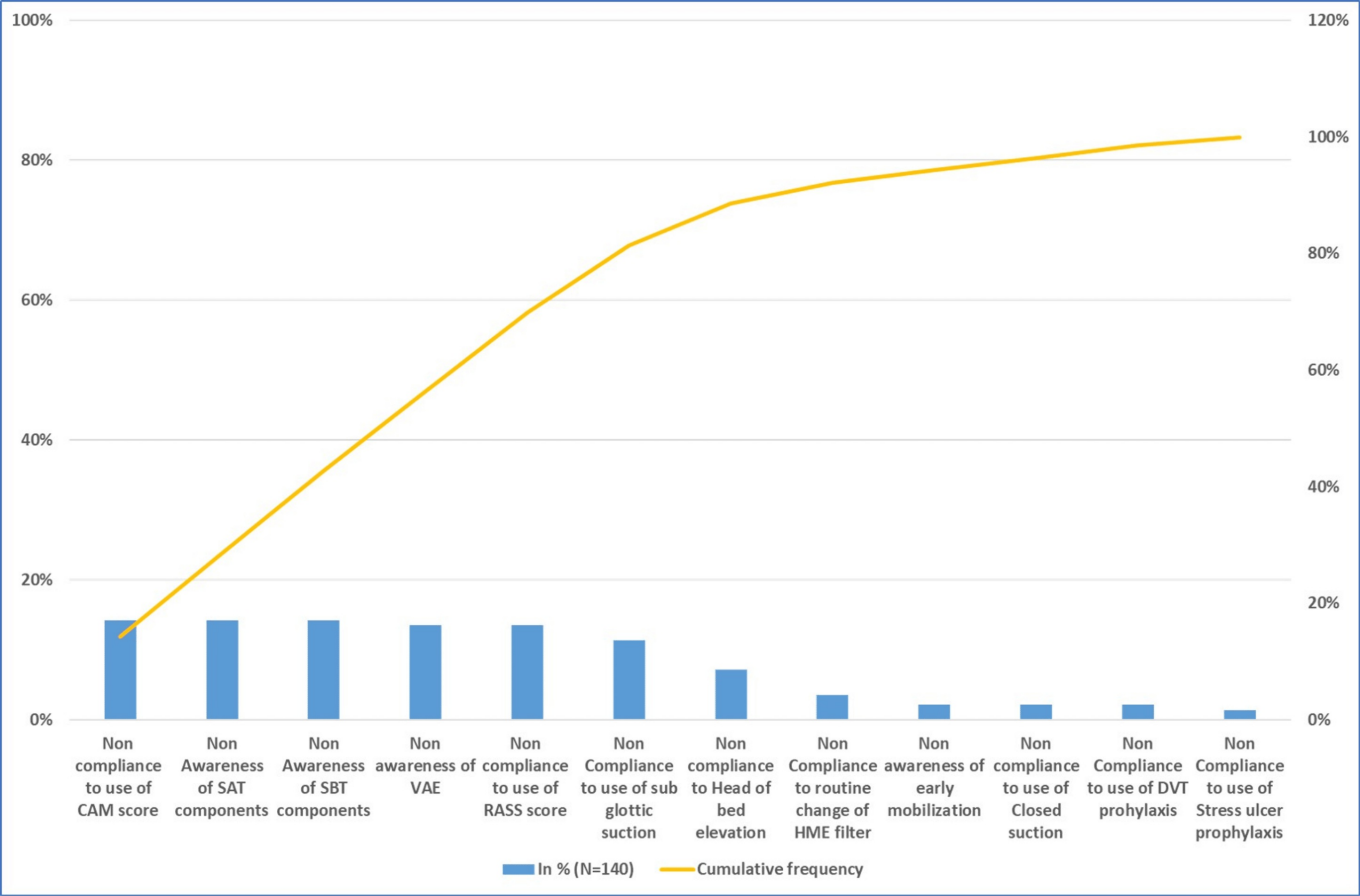
Pareto analysis of pre-pathway survey of ICU clinical staff The Pareto chart illustrates the distribution of barriers identified in a pre-implementation survey of ICU clinical staff (N=140) regarding ventilator-associated event (VAE) prevention practices. The blue bars represent the frequency of reported factors, while the overlaid yellow line depicts the cumulative percentage contribution of these factors to the total responses.

A pre-pathway survey of ICU staff (n=140) assessed baseline awareness and adherence to key components of the ventilator care bundle. This assessment identified existing gaps and informed pathway development. Pareto analysis revealed low baseline compliance and limited awareness across several critical elements of ventilator care (Figure 3). The most prominent gaps included non-compliance with CAM-ICU assessment, limited awareness of SAT and SBT, and inconsistent use of RASS assessments. Additional deficiencies were noted in subglottic suction use, head-of-bed elevation, and early mobilization practices. These findings highlighted the need for structured education and bedside training focused on these high-priority areas to improve adherence and overall VAE prevention practices, informing the development of the ventilator pathway.

Post-implementation compliance with the ventilator pathway

Initial compliance with the ventilator pathway was low, as reflected by the scorecard. Following educational sessions and implementation of the ventilator outcome tool, adherence improved markedly, reaching approximately 80-100% for most ventilator pathway components. This demonstrates that structured staff education and training, as well as systematic monitoring, are key to achieving and sustaining desired compliance and patient outcomes.

## Discussion

This retrospective study demonstrated a significant reduction in VAE (VAC, IVAC, PVAP) incidence and infection rates following implementation of a structured ventilator pathway in a tertiary care ICU. VAE incidence decreased from 28.46% in the pre-implementation period to 3.71% post-implementation (p<0.001), while total infection rates declined from 12.40% to 1.62% (p<0.001). These findings underscore the importance of ongoing staff education, bedside reinforcement, and standardized processes in optimizing adherence to evidence-based ventilator care practices and improving patient outcomes. The reduction in VAE incidence should be interpreted with caution, given the retrospective pre-post design, potential for unmeasured confounding, and absence of a concurrent control group; however, the observed reduction remains biologically plausible given the comprehensive, pathway-based intervention [9,23]. Our findings align with prior evidence showing that bundle-based interventions with educational reinforcement reduce VAP, VAE, and mechanical ventilation (MV) duration. Previous studies have similarly demonstrated that standardized surveillance and prevention strategies reduce ventilator-associated complications.

Our findings are consistent with existing evidence that indicates bundle-based interventions incorporating education, particularly when combined with structured education and compliance monitoring, lead to significant reductions in VAEs. A recent meta-analysis by Thapa et al. reported that care-bundle implementation in mechanically ventilated ICU patients leads to significant reductions in VAP and decreased MV duration by an average of 0.6 days when educational components are included [23]. Klompas et al. reported similar associations between bundle adherence and improved ventilator-related outcomes, emphasizing the importance of standardized surveillance and prevention strategies in reducing ventilator-related complications [9].

In the present cohort, approximately 40% of the admissions were medical and 60% surgical, with diabetes and hypertension being common comorbidities. However, no significant association was found between admission type or comorbidity burden and VAE incidence between the pre- and post-implementation groups, suggesting that the observed reduction in VAEs was unlikely to be driven by baseline patient differences. Disease severity, as measured by APACHE II scores, was also comparable between the two study groups, further supporting the conclusion that improvements in outcomes were attributable to changes in care processes rather than differences in illness acuity.

The overall hospital LOS differed significantly between the two groups, with a marked reduction observed in the post-implementation group; however, the ICU LOS and duration of MV did not differ significantly between the groups. The observed reduction in hospital length of stay, although modest (median reduction of three days), may have meaningful clinical and resource utilization implications in high-acuity ICU settings. Even small reductions in hospitalization duration can translate into decreased risk of hospital-acquired complications, including infections, improved bed availability and patient flow, and more efficient utilization of critical care resources, particularly in resource-constrained environments. Survival rates increased from 56.2% to 63.1% post-implementation of the ventilator pathway, but this difference did not reach statistical significance. While causality cannot be inferred from this observational design, these trends are consistent with prior studies demonstrating associations between improved ventilator care practices and downstream clinical outcomes [23,24].

Analysis of infection density suggested that prolonged MV was a key contributing factor to VAE development; the majority of mechanically ventilated ICU patients were affected by VAEs. This observation aligns with prior studies and suggests that cumulative exposure to ventilator support increases the risk of respiratory complications [2]. Analysis of VAE etiology revealed sepsis and septic shock as the most common causes of VAEs in our cohort, followed by ARDS, pneumonia, lung collapse, hypoxia, alveolar haemorrhage, and pneumothorax. These findings align with prior reports demonstrating that VAEs arise from both infectious and non-infectious causes, highlighting the need for prevention strategies beyond VAP alone [2,25].

The pre-implementation assessment of ICU staff revealed substantial gaps in awareness and adherence to ventilator care practices, including limited knowledge of VAE components, poor SAT/SBT awareness, inconsistent RASS and CAM-ICU assessments, underutilization of subglottic suction ETTs, and absence of a standardized process for care of mechanically ventilated patients in the ICU. These deficiencies informed the development of a structured ventilator pathway in our ICU, incorporating evidence-based care components, which was designed and implemented in accordance with AHRQ guidelines. Equal emphasis was placed on targeted staff education and compliance monitoring. Reinforcement strategies included bedside visual reminders, integration of a ventilator outcome tool for daily assessment and data capture, and ready availability of subglottic suction ETTs and tracheostomy tubes across all units.

Raising awareness and ensuring compliance with evidence‑based ventilator practices are critical for reducing complications, improving outcomes in mechanically ventilated patients who are at high risk for VAEs [23,24]. Accordingly, our initiative primarily emphasized improving staff awareness and standardizing care practices. Targeted education of the ICU team resulted in higher compliance and improved clinical outcomes. Implementation of the daily care process tool, incorporating subglottic suction drainage, structured sedation (RASS) and delirium (CAM-ICU) assessment, daily sedation vacation/SAT, weaning readiness assessment using a checklist, SBT administration, daily coordinated SBT/SAT, and early mobilization, improved adherence to best practices, facilitating effective weaning and reducing ventilator-dependent days.

Systematic monitoring, meticulous documentation, and multidisciplinary collaboration facilitated pathway implementation and consistent use of the daily care process tool. Ventilator data (daily minimum positive end-expiratory pressure (PEEP) and fraction of inspired oxygen (FiO2) values for patients ventilated for >48 hours) collection was optimized through collaboration among critical care physicians, nurses, infection preventionists, and respiratory therapists. Maintaining a ventilator pathway compliance checklist and an adherence scorecard enabled real-time performance tracking, which revealed 80-100% compliance across most ventilator pathway components and helped achieve daily mobility goals specifically designed to enhance recovery. Initial lower compliance (61-80%) with delirium assessment prompted the introduction of a delirium-prevention bundle and a bedside retraining program, resulting in measurable subsequent improvements. Continuous VAE surveillance facilitated assessment of pathway efficacy.

Effective interdisciplinary communication and teamwork are essential for sustaining patient-centered care in the ICU, particularly for mechanically ventilated patients [26]. Structured strategies, including regular interdisciplinary meetings, bedside rounds, alignment of care plans with patient goals/values/preferences, and targeted staff training (care principles, cultural sensitivity, and communication skills) have been shown to enhance communication, coordination, and overall quality of care [26-28]. Building on these principles, weekly training sessions, including workshops led by respiratory therapists and intensivists, were conducted to enhance awareness and reinforce compliance. Performance review by ICU nurse managers ensured adherence, while regular meetings of the critical care improvement committee were held to review progress, identify barriers, and implement corrective actions. This structured educational and feedback approach contributed to sustained compliance and improved the quality of care delivered to mechanically ventilated patients. Consistent with previous reports, higher pathway adherence correlated with improved outcomes [23,29].

This study has several limitations. Its single-centre, retrospective before-and-after design limits generalizability and may preclude definitive causal inference. The absence of a concurrent control group introduces potential for temporal confounding. Unequal group sizes and lack of multivariable adjustment may have influenced the observed associations. Additionally, unmeasured changes in ICU practices, staffing, or infection control measures during the study period may have contributed to the observed improvements; however, these factors cannot be fully accounted for in a retrospective pre-post design. Nevertheless, the strength of this study lies in the structured, pathway-based quality improvement intervention that was evaluated in a real-world ICU setting, demonstrating sustained reductions in ventilator-associated events with systematic monitoring and multidisciplinary implementation.

Implementation of the ventilator pathway was associated with a marked reduction in VAE incidence and a favorable trend in survival, suggesting that the adopted strategies were effective in reducing ventilator-related complications. Successful implementation required collective coordinated engagement of the ICU team, enhanced interdisciplinary communication, meticulous care planning, and establishing clearly defined care standards. Real-time compliance monitoring identified protocol deviations, enabling timely corrective measures. This contributed to progressive improvements in staff adherence and overall ventilator care quality, ultimately leading to reduced incidence of VAEs and associated risks.

## Conclusions

The structured educational and interventional program implemented in the present study was associated not only with improved adherence to ventilator care practices but also with significantly decreased VAE rate among mechanically ventilated ICU patients. These improved outcome metrics underscore the value of clinical pathways as viable and effective tools for attaining sustainable quality improvement in healthcare and improving patient safety. Further multicenter studies with larger sample sizes are needed to validate these findings. Ongoing education, surveillance, and reinforcement regarding ventilator safety initiatives among healthcare personnel remain essential to mitigating VAE incidence and associated mortality.
